# Impact of cognitive biases on environmental compliance risk perceptions in international construction projects

**DOI:** 10.3389/fpsyg.2024.1397306

**Published:** 2024-11-21

**Authors:** Tengyuan Chang, Yuxuan Du, Xiaopeng Deng, Xianru Wang

**Affiliations:** ^1^Institute of Human Rights, Law School, Southeast University, Nanjing, China; ^2^China-Pakistan Belt and Road Joint Laboratory on Smart Disaster Prevention of Major Infrastructures, Southeast University, Nanjing, China

**Keywords:** environmental protection, corporate compliance, cognitive biases, risk management, international construction projects

## Abstract

**Introduction:**

This research explores the complex interplay between cognitive biases and Environmental Compliance Risk Perception (ECRP) in international construction projects. Understanding such a relationship is essential as it can have significant implications for the success and environmental sustainability of these projects.

**Methods:**

This study analyzed a scenario-based questionnaire survey conducted with 270 international construction practitioners. It employed Partial Least Squares Structural Equation Modeling (PLS-SEM) to identify the influencing factors of cognitive biases and their impact on ECRP. The factors considered included individual, organizational, and project-specific aspects.

**Results:**

The analysis revealed that Cultural Bias (CuB), Confirmation Bias (CoB), and Short-Termism (ST) significantly influence ECRP. These biases are affected by a complex interplay of the aforementioned antecedents.

**Discussion:**

Based on the results, an innovative framework for evaluating ECRP was proposed. Additionally, a management strategy was developed to recognize and mitigate the cognitive biases of professionals during the bidding and execution phases of international construction projects. By clarifying the factors influencing cognitive biases and their complex relationship with ECRP, this research emphasizes the importance of addressing employee cognitive biases in conjunction with improving environmental awareness in environmental compliance management. It fills a crucial gap in the existing literature and offers international contractors strategies to reduce these biases, thereby enhancing their environmental protection capabilities and minimizing potential negative environmental impacts from international construction projects, which is vital for advancing sustainable development.

## 1 Introduction

Environmental protection is a pivotal concern in international construction projects (Chen et al., 2016). International contractors must rigorously adhere to standards that safeguard the environment, ecology, and human health. In these projects, contractors face the challenge of ensuring environmental compliance within complex legal and regulatory frameworks. Compliance risk refers to the negative consequences that businesses may face for failing to adhere to laws, norms, business ethics, and international agreements (Gupta et al., 2019). These risks can lead to legal penalties, financial losses, and reputational damage, and may further result in project disruptions, market share decline, and loss of investor confidence (Cantele and Zardini, 2020; Wang et al., 2021; Yin et al., 2023). Therefore, it is crucial for international contractors to implement effective environmental compliance management to mitigate these compliance risks.

The essence of environmental compliance management is to ensure that all levels of an organization—from frontline workers to management and stakeholders—have a profound commitment to environmental responsibility and that their actions fully adhere to the highest legal, ethical, and societal standards for environmental protection. In this system, frontline employees, as direct implementers of environmental compliance measures, play a crucial role (Chi and Yang, 2024). Their daily operations and decision-making directly determine whether the organization adheres to environmental regulations and standards, thus impacting the effectiveness of compliance measures (Chang et al., 2024; Daily and Huang, 2001). Raising employee awareness of the importance of environmental protection is foundational to achieving organizational goals for environmental compliance management. However, despite recognition of this by many companies, achieving uniform compliance behavior across the workforce remains a significant challenge. Even with standardized policies in place, individual differences in environmental compliance behavior continue to be a persistent issue (Okumah et al., 2018; Zsóka, 2008). In practice, many projects or companies tend to prioritize the development of compliance procedures over fostering a deep commitment to environmental responsibility among their staff (Perron et al., 2006). Nevertheless, the importance of individual characteristics in influencing corporate compliance management is increasingly recognized (Graafland, 2020; Schaefer et al., 2020), and a lack of awareness about environmental protection often leads to non-compliance (Hwang and Tan, 2012). Therefore, a deep understanding of the factors that influence individual environmental compliance is crucial for strengthening the environmental compliance framework of a company.

Environmental Compliance Risk Perception (ECRP) involves an individual’s recognition and evaluation of the potential negative consequences arising from non-adherence to environmental regulations. This perception significantly influences whether individuals can recognize the potential threats and consequences of non-compliant behaviors (Slovic and Peters, 2006), and decisively impacts their actions (Zhang et al., 2020). A strong ECRP typically prompts individuals to act cautiously and adhere strictly to environmental regulations (Fritsche et al., 2010), thus preventing potential environmental damage. Conversely, underestimating these risks may lead to neglect of environmental compliance. Empirical evidence indicates that heightened awareness of ECRP is associated with improved risk identification and control capabilities (Deka et al., 2023). However, even individuals with a profound understanding of environmental compliance may engage in non-compliant behaviors due to cognitive biases that distort their assessment of environmental risks in certain contexts (Sörqvist and Langeborg, 2019). This suggests that even high environmental awareness does not necessarily prevent judgment errors introduced by cognitive biases.

International construction employees face significant environmental compliance challenges arising from complex, multicultural, and legal environments (Kim and Nguyen, 2021), further complicated by frequent transient or short-term work arrangements (Luo et al., 2023). Such conditions may exacerbate cognitive biases, leading individuals to believe they are less vulnerable to negative environmental outcomes compared to others (Johnson and Levin, 2009), which, in turn, diminishes their ECRP and increases the likelihood of non-compliant behavior. Therefore, comprehending how cognitive biases affect individuals’ ECRP, and addressing these biases, is essential for improving environmental compliance management in international construction projects.

In international construction projects, scholarly research on environmental compliance has primarily focused on developing macro-level policies, applying relevant technologies, and implementing corporate management strategies (Luo et al., 2022; Tam et al., 2006; Zutshi and Creed, 2015). These studies aim to establish and rigorously evaluate transnational environmental compliance programs, manage environmental legal and ethical risks within projects, and identify factors that lead to non-compliant behaviors. Existing research has explored the impact of organizational structures (Atkinson et al., 2000), regulatory frameworks (Gouldson and Murphy, 2013; Karplus et al., 2021), external environments (Dasgupta et al., 2000; Rorie, 2015), and industrial standards (Heyl et al., 2021) on both corporate and individual environmental behaviors. Despite a deep understanding of the systemic and strategic aspects of environmental compliance, studies on how individual employees influence environmental compliance are still relatively scarce. While some research has begun to examine the relationships between personal cognition, emotions, attitudes, and ethical decision-making with corporate compliance behaviors (Dasanayaka et al., 2022; Fineman and Sturdy, 1999; Liao, 2016), the specific role of cognitive biases in affecting individuals’ ECRP in particular contexts still requires further exploration.

This gap in research has motivated the current study to delve deeper into the cognitive biases influencing international construction employee’ ECRP. This study aims to uncover the influencing factors of these biases and develop effective interventions to mitigate their impact. In doing so, this research seeks to enhance the accuracy of international contractors’ ECRP. This scholarly effort not only reveals the complex mechanisms of cognitive biases in environmental compliance decision-making but also provides innovative strategies and insights for improving environmental risk assessment and management practices in international construction projects. This study is expected to enhance the capabilities of international contractors in environmental compliance management, thereby mitigating the negative impacts of construction activities on environment.

## 2 Theory and hypotheses

### 2.1 Individual factors in environmental protection

In the realm of environmental protection, the study of individual behavioral factors has increasingly become a focal area, demonstrating that elements such as personal environmental awareness, emotions, and attitudes play an indispensable role in advancing corporate environmental management (Dasanayaka et al., 2022). The significance of individual factors, especially environmental consciousness, cannot be understated. Such awareness not only motivates individuals to adopt more environmentally friendly behaviors but also facilitates the development and implementation of eco-friendly policies within organizations (Blackstock et al., 2010; Okumah et al., 2018). However, international construction projects often involve complex multicultural interactions, legal and regulatory environments, and dynamic working conditions (Luo et al., 2022). In these contexts, employees may develop cognitive biases based on limited information or specific situational influences, affecting their understanding and adherence to environmental regulations (Sörqvist and Langeborg, 2019). These biases can result in a misalignment between employees’ existing environmental awareness and the environmental compliance requirements of the project’s location. Therefore, in environmental compliance management, corporations must not only strive to improve employees’ environmental awareness but also address the effects of cognitive biases.

### 2.2 Cognitive bias in risk perception

The increasing scrutiny on cognitive biases has highlighted their critical role in influencing individuals’ perceptions of risk. Research suggests that the bounded nature of human cognition constrains the ability to exhaustively search and precisely interpret information (Cooper and Artz, 1995). As a result, individuals often resort to heuristic thinking—a mental shortcut—to expedite decision-making. This intuitive approach can predispose individuals to cognitive biases (Schwenk, 1986), systematically deviating from rational thought processes in both pattern and prevalence (Baron, 2018). Sitkin and Pablo (1992) observed that such biases become more pronounced in environments characterized by complexity and uncertainty. In the context of compliance risk, time constraints or information overload may lead individuals or organizations to rely on heuristic thinking, causing oversight regarding the applicability and universality of rules. This, in turn, can result in an underestimation or overestimation of the consequences of non-compliant behaviors (Riza, 2015). Therefore, exploring how cognitive biases affect the ECRP can provide a novel theoretical perspective for a more accurate understanding of the intrinsic reasons behind individual non-compliance.

Cognitive biases, in their multifaceted appearances (Baron, 2023), profoundly affect the environmental compliance landscape of international construction projects, where employees confront the complex cross-cultural exchanges (Ochieng and Price, 2010), a variety of regulatory standards, and the constant pressures of deadlines and budgets (Kim et al., 2009). In such environments, Cultural Biases (CuB), Confirmation Bias (CoB), and a tendency towards Short-Termism (ST) markedly skew perceptions of compliance risks. CuB stems from socialization, leading individuals to unconsciously interpret actions and norms through their own cultural framework (Triandis, 2006). CoB indicates a predilection in processing information that favors existing beliefs, prompting individuals to selectively seek out, process, and recall information that reinforces their preconceptions, while neglecting disconfirming data (Nickerson, 1998), whether encountered in the external environment (Hart et al., 2009) or retrieved from memory (Gurcay-Morris, 2016). ST disproportionately focuses on immediate outcomes at the cost of long-term consequences, especially in high-pressure situations (Zimbardo and Boyd, 2014).

Cognitive biases are shaped by a complex interplay of psychological elements and situational factors at the individual cognitive level. These factors encompass a wide range from personal experiences, habits, and cognitive styles to memory mechanisms, emotional states, cultural backgrounds, and situational pressures (Kahneman, 2011). Cognition is not only individualistic but also social in nature, meaning that cognitive processes can be influenced by elements like political systems, social culture, media, and interpersonal interactions (Bertoldo et al., 2021; Joffe, 2003). In international construction projects, employees often find themselves in a temporary and independent project environment, where their social interactions are primarily related to project and organizational stakeholders. Consequently, their cognitive biases are not solely determined by personal factors but are profoundly influenced by project-specific and organizational factors. This influence manifests through aspects such as Multicultural Background (MCB), Project Complexity in Uncertainty (PCU), Time Pressure (TP), Organizational Culture-Driven Leadership (OCL), International Experience Level (IEL), Resource-Constrained Project Scale (RCPS), and Efficient Communication Flow (ECF).

### 2.3 Hypotheses development

#### 2.3.1 Multicultural background, MCB

MCB influences communicative practices, workplace norms, and the interpretation of environmental compliance standards, thus shaping ECRP. Culture plays a crucial role in forming individual identities, guiding their cognitive processes, and directing their behaviors (Kitayama and Park, 2010). An individual’s sociocultural environment profoundly affects their perspectives and cognitive approaches (Ji et al., 2001). Within intercultural settings, inherent cultural frameworks may predispose individuals to CuB, impacting their comprehension and management of compliance challenges (Triandis, 2006). This issue is particularly pertinent in international construction projects, where team members’ diverse cultural backgrounds can alter their interpretation of environmental compliance, leading to different risk assessments. Hence, the study posits the following hypothesis:

Hypothesis 1: MCB significantly positively influence CuB.

#### 2.3.2 Project complexity in uncertainty, PCU

In international construction projects, PCU compounds the challenges of data interpretation and escalates the ambiguity inherent in decision-making (Milliken, 1987). In such a context of complexity and increased uncertainty, employees often find that cognitive heuristics, a form of intuitive thinking, are more suitable and effective than comprehensive analytical methods (Butler et al., 2014). Thus, when grappling with complex environmental compliance information, there is a propensity among employees to default to intuition and heuristic-based judgments (Inbar et al., 2010; Roghanizad and Neufeld, 2015). Yet this predilection can lead astray, particularly when preconceptions steer the cognitive process, culminating in CoB—the selective search for, interpretation of, and memory for information that aligns with existing beliefs, while contrary evidence is disregarded or diminished (Tversky and Kahneman, 1974). Accordingly, this study advances the following hypothesis:

Hypothesis 2: PCU significantly positively influences CoB.

#### 2.3.3 Time pressure, TP

International construction projects, known for their expansive scale and tight deadlines, place immense pressure on employees to make prompt decisions and actions. TP, defined as the subjective experience of insufficient time for task completion (Lallement, 2010), often triggers a spectrum of stress-related responses. This sense of urgency can limit thoughtful reasoning and logical processing (Evans and Curtis-Holmes, 2005), pushing individuals toward rapid, intuition-based decisions. Constrained by such pressure, decision-makers may exhibit a bias towards the immediate, phenomenon termed ST, which often results in overlooking wider, long-term implications (Keough et al., 1999). Within the demanding context of international construction, this bias manifests in a predilection for expedient solutions in environmental compliance-related decisions (Young et al., 2012), potentially at the expense of environmental compliance (Graafland, 2016; Slawinski et al., 2017). This leads to the articulation of the following hypothesis:

Hypothesis 3: TP significantly positively influences ST.

#### 2.3.4 Organizational culture-driven leadership, OCL

In international construction project management, the intertwined dynamics of OCL are critical. Organizational culture, representing the collective values of a team, develops through addressing external challenges and fostering internal growth, which shapes the behaviors, beliefs, and principles within the team (Schein, 1992). Leadership style, reflecting the attributes and actions of leaders, significantly influences team cognition and decision-making processes (Peterson, 1997). These cultural and leadership determinants act as a cohesive framework that can diminish biases arising from cultural diversity and enhance intercultural collaboration (Vaara et al., 2012). Clearly defined norms, along with leadership that appreciates diversity and prioritizes strategic foresight, are instrumental in mitigating biases toward confirming existing beliefs and resisting the lure of short-term gains (Scott and Bruce, 1995). This organizational climate cultivates a consideration for a full spectrum of information, urging a balance between immediate results and long-term outcomes, thus fostering objective cognition amidst diverse cultural backgrounds (Marchisotti et al., 2018). Therefore, this study posits the following hypotheses:

Hypothesis 4a: OCL significantly negatively influence CuB.

Hypothesis 4b: OCL significantly negatively influence CoB.

Hypothesis 4c: OCL significantly negatively influence ST.

#### 2.3.5 International experience level, IEL

Individuals steeped in international experiences often outperform in cultural intelligence and adaptability, which enhance their ability to accurately interpret behaviors and norms across diverse cultural contexts (Earley and Ang, 2003). Such proficiency is crucial for minimizing CuB. In contrast, employees with limited international exposure may struggle to identify compliance risks. Their lack of familiarity with relevant regulations leads to interpretative errors. Additionally, IEL profoundly affects information-processing strategies. Faced with complex compliance challenges, experienced individuals are inclined to critically evaluate, and question existing information, integrating their understanding of different regulatory environments and their informed perspectives to assess information with greater objectivity (Low et al., 2019), thus reducing susceptibility to CoB. On this basis, the following hypothesis is presented:

Hypothesis 5a: IEL significantly negatively influence CuB.

Hypothesis 5b: IEL significantly negatively influence CoB.

#### 2.3.6 Resource-constrained project scale, RCPS

Large-scale projects in the realm of international construction, particularly those with significant implications for environmental compliance, are known for their enhanced complexity and substantial resource demands (Onubia et al., 2019). These projects present significant time management and resource allocation challenges for project teams, who also contend with investor pressures and financial constraints (Zhang and Gimeno, 2016). Research indicates that increased project dimensions and tight resource allocation can lead to a rise in noncompliance, with large-scale efforts especially susceptible to concealing environmental violations and exaggerating environmental claims (Geraldi et al., 2011). Confronted with strict deadlines and resource shortages, teams might lean towards Short-Termism (ST), foregoing long-term environmental sustainability objectives for immediate gains to swiftly meet client demands (Souder et al., 2016). The expansion in project scale and reduction in resources result in a depletion of spare capacity among employees, heightening their exposure to complex environmental information (Kleinknecht et al., 2020). When cognitive resources become strained, teams may inadvertently focus on short-term requirements, neglecting the critical aspects of ongoing environmental compliance and project integrity.

Hypothesis 6: RCPS positively influence ST.

#### 2.3.7 Efficient communication flow, ECF

Effective communication acts as the lifeline for information transfer and exchange in international construction projects (Darma and Supriyanto, 2017). When information flows through an organization’s levels, any hierarchical or incomplete messaging can significantly disrupt decision-making, potentially leading to choices based on partial insights. Therefore, the fluidity of communication and the streamlining of information flow are essential to empower project teams with the timely and accurate assimilation of compliance-centric data. Clear internal communication not only defines compliance responsibilities for employees but also highlights improvement areas, which is necessary for bridging cultural divides and mitigating biases in international contexts. Given that cognitive biases are inherent in human judgment (Joslyn et al., 2021), their intensification amid communication deficits or limited information pathways is significant. Well-managed information flow facilitates access to varied viewpoints and comprehensive data (Broom, 2005), reducing the impact of biases, particularly when confronting deeply held beliefs. Drawing from these observations, the following hypotheses are formulated:

Hypothesis 7a: ECF significantly negatively affect CuB.

Hypothesis 7b: ECF significantly negatively affect CoB.

#### 2.3.8 Cognitive bias

Ideally, individuals would uniformly perceive risk across similar scenarios (Nutt, 1986). However, when faced with complex and ambiguous issues, there’s a human tendency to rely on effortless intuitive cognition rather than strenuous logical reasoning, known as the ‘cognitive miser’ effect (Stanovich, 1999), which fosters irrationality and incompleteness in cognitive operations. Research reveals that cognitive biases skew focus and interpretation of information, often leading to an underplayed awareness of potential negatives and uncertainties in decision-making, thereby dampening perceived risks (Barnes, 1984; Măirean et al., 2022; Simon et al., 2000). In the specialized setting of international construction projects, the influences of CuB, CoB, and ST are particularly pertinent in environmental compliance-related decisions. CuB can lead to a misinterpretation of environmental compliance within unfamiliar settings, downplaying potential risks. CoB may drive employees to heed only confirmatory information, thus narrowing their ECRP. Influenced by ST, individuals tend to make optimistic risk assessments hastily, overlooking the long-term consequences of environmental compliance. From these insights emerge the following hypotheses:

Hypothesis 8a: CuB significantly negatively affects ECRP.

Hypothesis 8b: CoB significantly negatively affects ECRP.

Hypothesis 8c: ST significantly negatively affects ECRP.

**Figure 1:**
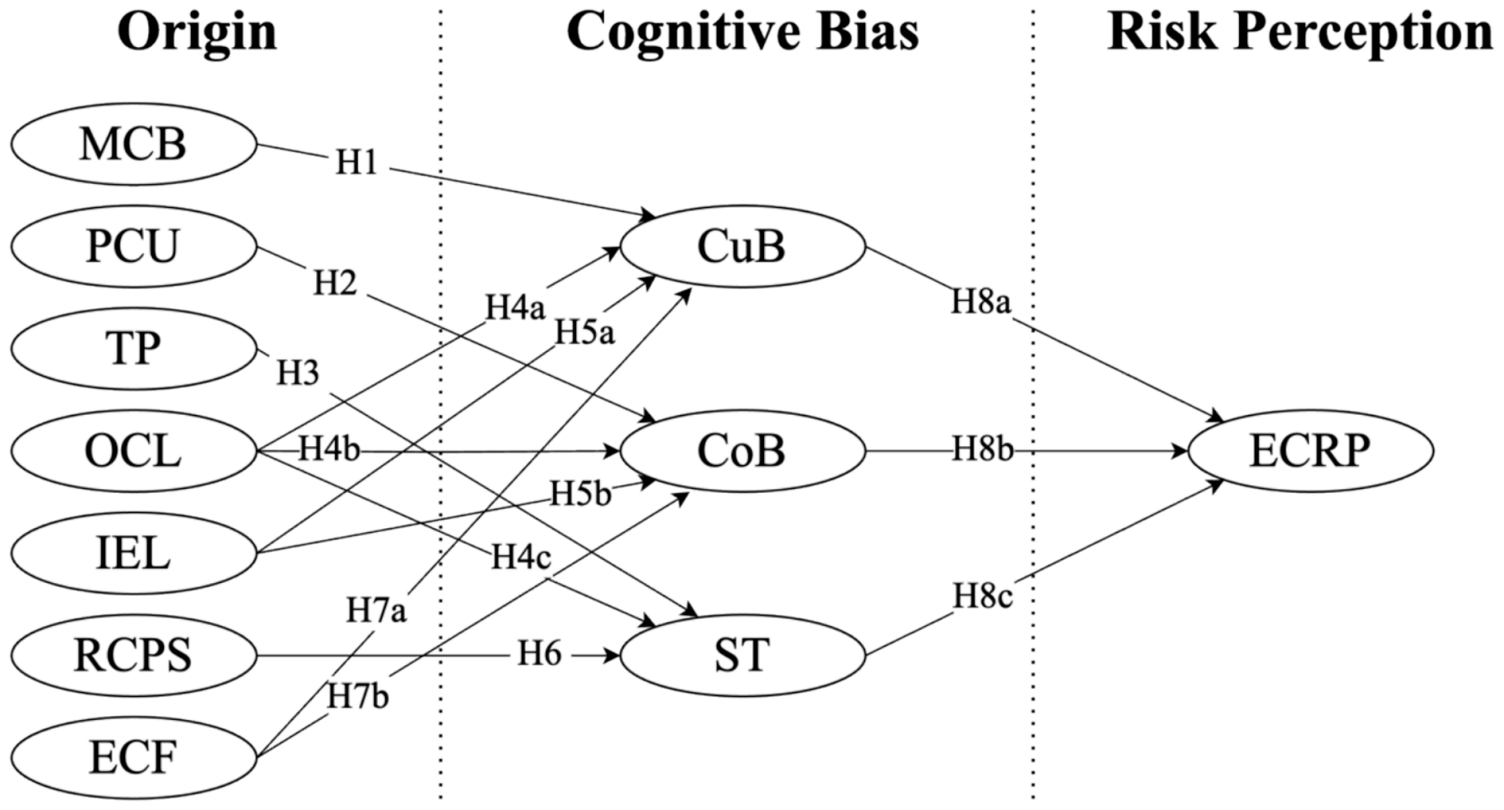
Hypothesis model.

All the hypotheses are displayed in Figure 1.

## 3 Methods

### 3.1 Sample and data collection

Given the subjective nature of the variables within the model, this study employed a questionnaire survey as the main data collection method. To address the sensitivity and covert aspects of the topics covered in the questionnaire, three specific strategies were implemented to mitigate defensive responses from participants (Brown and Loosemore, 2015). Firstly, to alleviate any potential concerns or apprehensions among participants, the study clarified that the questionnaire served an academic purpose, seeking responses based solely on personal perceptions without the constraint of right or wrong answers. Secondly, the respondents were assured of complete anonymity in the survey, guaranteeing that no personal information or survey results would be disclosed. Finally, a hypothetical scenario approach was adopted, where respondents were prompted to respond to scenarios rather than actual events, a technique designed to reduce the influence of social desirability bias (Hofeditz et al., 2017; Hong and Furnell, 2021).

To ensure that the survey scenarios were comprehensible to respondents, this study initially identified common compliance situations from case studies in the field of international construction projects. Interviews with five professionals, experienced in international construction projects, subsequently helped in selecting the most relevant scenario for the questionnaire. Additionally, to guarantee the validity and reliability of the adopted hypothetical scenarios, this study provided detailed descriptions of these scenarios, and validated them through expert review and pilot testing. The expert panel, composed of senior professionals from the field of international construction projects, assessed the practical relevance and comprehensiveness of the scenarios. The pilot test was conducted among a small group of the target audience to check for understanding and response consistency of the scenarios, ensuring the reliability and accuracy of the measurement results. The finalized scenario is as follows.

Imagine you are a key member of an international construction project spanning multiple countries, with a focus on environmental compliance. The project team consists of international construction employees from diverse cultural backgrounds, each bringing their unique understanding and practices of environmental compliance. The transnational nature of the project demands navigating through complex and varying environmental laws and regulations, along with strict challenges in time and resource allocation. In this context, the success of the project hinges not only on technical and managerial competencies but also on effective cultural communication and a firm commitment to environmental compliance. Facing this international construction project, especially considering its relevance to environmental compliance, please respond to the following compliance-related questions based on what you perceive as the most likely scenario.

Given the uncertain number of expatriates in international construction projects, a snowball sampling method was utilized to ensure a statistically robust sample. Initial participants, who were familiar with the researcher’s network were sourced from various organizations like the China International Contractors Association. These participants were also encouraged to refer other colleagues who might be interested in the survey.

### 3.2 Measures

The questionnaire was initially developed based on a comprehensive review of relevant literature and further tailored to suit the specific context of environmental compliance in international construction projects. To ensure the questionnaire’s validity and reliability, a small-scale pilot study was conducted involving 10 experts from the field of international construction, including 5 executives from global firms and 5 academic scholars in construction management. These experts completed the draft questionnaire and provided feedback on the clarity, relevance, and design of the questions, helping to identify and rectify any issues that could impact the validity of the responses. Based on this feedback, minor adjustments were made to the questionnaire, such as rephrasing questions for greater clarity, adjusting the scale of certain items, and removing redundant questions. These modifications were confirmed after a secondary review by the expert panel. Subsequently, 20 Employees with extensive experience in international construction were chosen to further test the questionnaire to ensure its content, structure, and length were suitable for formal distribution. They were requested to complete the questionnaire to validate its appropriateness, relevance, and clarity. The final questionnaire was divided into three parts: (1) an explanation of the environmental compliance scenarios; (2) questions related to personal information, such as years of work experience; and (3) an assessment of the relevant items based on the respondents’ experience, using a Likert scale ranging from 1 (strongly disagree) to 5 (strongly agree) (Table 1).

**Table 1:** Scale items.

Category	Items	References
MCB	The project team is culturally diverse.	Shenkar et al. (2008)
	Significant cultural differences exist within the project team.	
	Problems should be addressed with a multicultural approach.	
PCU	The project may encounter numerous environmental protection challenges.	Zulu et al. (2022)
	Project goals and requirements frequently shift.	Perminova et al. (2008)
	Unexpected challenges are common throughout the project.	
TP	The project may have strict deadlines.	Maruping et al. (2015)
	There is intense time pressure to complete the project tasks.	
	Long-term planning is complicated by the project’s urgency.	Silayoi and Speece (2004)
OCL	Leadership promotes innovation and risk-taking in the project.	Wu et al. (2022)
	Leadership encourages proactive, innovative compliance approaches in the project.	Santos et al. (2012)
	Compliance is a core value and responsibility in this organization.	Weaver et al. (1999)
IEL	Team members have extensive international construction experience.	Dow and Larimo (2011)
	Team members are familiar with global environmental compliance standards.	Earley and Ang (2003)
	Team members boast ample expertise.	Bird and Osland (2004)
RCPS	The project is large in scale.	Nassar and Hegab (2006)
	Resource allocation often affects the environmental protection of the project.	Cheng and Kesner(1997)
	Resource shortages are frequently encountered during the project.	Dao et al. (2016)
ECF	Information exchange within the team is efficient.	Citroen (2011)
	Quick dissemination of crucial details is ensured.	Durugbo et al. (2013)
	Team members demonstrate high efficiency in information exchange.	Diallo and Thuillier (2005)
CuB	In this context, cultural differences lead employees to develop biased understandings of environmental compliance risks.	RIPPL (2002)
	In this context, cultural diversity makes it difficult for employees to reach consensus on environmental compliance standards.	Johnson and Swedlow (2019)
	In this context, cultural diversity causes employees to have differing perspectives and approaches when evaluating and responding to environmental compliance risks.	RIPPL (2002)
CoB	In this context, employees tend to focus only on environmental compliance information that aligns with their existing beliefs.	Wickens and Hollands (2000)
	In this context, environmental compliance information that contradicts employees’ expectations is often ignored or underestimated.	Jonas et al. (2001)
	In this context, evidence that challenges employees’ assumptions about environmental compliance risks is often overlooked.	Jonas et al. (2001)
ST	In this context, employees may overlook long-term environmental compliance risks.	Murphy (2004)
	In this context, employees’ focus on immediate project outcomes overshadows the need for long-term environmental planning	Marginson and McAulay (2008)
	In this context, employees’ attention to short-term gains may lead to neglect of long-term environmental compliance risks.	Bushee (2001)
ECRP	In this context, employees fail to effectively identify environmental compliance risks in the project.	Roberto (2002)
	In this context, employees lack a sufficient understanding of environmental compliance risks.	
	In this context, employees do not allocate enough attention and resources to managing environmental compliance risks.	

### 3.3 Characteristic of respondents

The survey was administered online using the Wenjuanxing platform. Out of the 275 questionnaires collected, 5 were deemed invalid due to incomplete responses or clear errors, such as uniform responses across all items. This resulted in a total of 270 valid responses, representing a diverse range of demographic characteristics (Table 2). Work experience among participants ranged considerably, with a minor segment (7.78%) having less than 3 years, 17.78% possessing 3–5 years, a substantial proportion (31.48%) encompassing 6–10 years, followed by 24.81% with 11–15 years, and a notable 18.15% exceeding 15 years of experience. Regarding the professional hierarchy, the sample included 15.56% senior managers, 10.37% department managers, and 20.74% project managers, with engineers forming the majority at 43.7%, and the remainder at 9.63% falling into other categories. Geographical representation was extensive, with 31.48% of respondents from China, an identical proportion from other Asian countries (excluding China), 18.15% from Africa, 5.19% from Europe, 2.96% from North America, 4.07% from South America, and 6.67% from Australia, thus ensuring a globally diverse perspective.

**Table 2:** Demographic characteristics of respondents.

Characteristics	Items	Frequency (*n* = 270)	Percentage
Work experience	<3	21	7.78
	3–5	48	17.78
	6–10	85	31.48
	11–15	67	24.81
	>15	49	18.15
Rank	Senior manager	42	15.56
	Department manager	28	10.37
	Project manager	56	20.74
	Engineer	118	43.7
	Else	26	9.63
Area	China	85	31.48
	Asia (except China)	85	31.48
	Africa	49	18.15
	Europe	14	5.19
	North America	8	2.96
	South America	11	4.07
	Australia	18	6.67

### 3.4 Data analysis approach

First, we performed descriptive statistical analysis on the collected questionnaire data using SPSS software. Following this, Structural Equation Modeling (SEM) was employed as a key analytical tool to explore and validate the complex interrelationships among the various theoretical constructs. Given the limitations of non-normal data and a small sample size, Partial Least Squares SEM (PLS-SEM) was chosen for its suitability (Hair et al., 2011), with a sample size of 270 proving to be sufficient for robust analysis. Data analysis was conducted using Smart PLS 3, employing a path weighting scheme and setting the iteration maximum to 300 (Hair et al., 2021). A bias-corrected and accelerated bootstrapping approach with 5,000 subsamples was chosen for accuracy. To address common method bias, Harman’s one-factor test was applied (Podsakoff et al., 2003), revealing that the largest factor explained only 31.45% of the variance, significantly below the 50% threshold (Podsakoff and Organ, 1986), suggesting minimal bias. Confirmatory tests comparing early and late participant responses showed no significant differences, affirming the survey’s reliability.

## 4 Results

### 4.1 Descriptive statistical analysis

First, the scale reliability of the data from 270 valid questionnaires was assessed. Based on the analysis using SPSS software, the Cronbach’s alpha coefficient for the questionnaire data was 0.908 (>0.700), indicating a high level of reliability. Additionally, we calculated the mean values for the three cognitive biases and ECRP. The results showed that the mean values for CuB, CoB, and ST were 3.54, 3.72, and 3.97, respectively, suggesting that these cognitive biases are prevalent among international construction employees, with an overall moderate to high level, and ST being the most prominent. The mean ECRP score was 3.32, indicating that respondents’ perception of environmental compliance risks was generally moderate to weak. Furthermore, a Kruskal-Wallis test, a common non-parametric test, was conducted to examine whether there were significant differences in the scores for 33 variables across respondents with different levels of experience or positions. The results showed that all asymptotic significance values were well above 0.05 (Siegel and Castellan, 1988), indicating no significant differences among respondents with varying backgrounds.

### 4.2 Measurement model

Table 3 illustrates the robustness of the measurement model. Cronbach’s alpha values for the latent variables, ranging from 0.718 (RCPS) to 0.832 (CoB), all exceed the recommended threshold of 0.7, indicating strong internal consistency (Hair et al., 2019). The Average Variance Extracted (AVE) values, spanning from 0.629 (CoB) to 0.727 (IEL), surpass the standard minimum of 0.5, demonstrating substantial convergent validity (Hair et al., 2019). Factor loadings for these constructs, between 0.727 (CuB) and 0.913 (TP), all exceed the 0.7 benchmark, confirming the appropriateness of the items for their respective constructs. Composite Reliability (CR) values, ranging from 0.836 (CoB) to 0.889 (IEL), further affirm the reliability and consistency of the constructs. These values, along with the high AVEs, indicate that the constructs reliably capture the variance of their indicators. Discriminant validity is also established, as the square roots of the AVEs for each construct are significantly greater than their respective inter-variable correlations (Henseler et al., 2015). This assures that each construct is distinct and captures unique variance. The maximum loadings of each variable on its respective construct, as shown in Table 4, confirm the validity of the theoretical model (Chin, 1998), ensuring the measurement model’s reliability, convergent validity, and discriminant validity for further analysis.

**Table 3:** Factor loadings, composite reliability, and internal consistency.

Variables	Factor loading	AVE	CR	Cronbach’s alpha
MCB	0.738–0.891	0.641	0.842	0.782
PCU	0.750–0.843	0.651	0.848	0.793
TP	0.781–0.913	0.690	0.869	0.805
OCL	0.742–0.856	0.639	0.841	0.810
IEL	0.835–0.891	0.727	0.889	0.823
RCPS	0.768–0.901	0.698	0.873	0.718
ECF	0.806–0.915	0.716	0.883	0.742
CuB	0.727–0.856	0.646	0.845	0.779
CoB	0.763–0.829	0.629	0.836	0.832
ST	0.744–0.892	0.652	0.848	0.784
ECRP	0.785–0.853	0.679	0.864	0.803

**Table 4:** Result of discriminant validity.

	MCB	PCU	TP	OCL	IEL	RCPS	ECF	CuB	CoB	ST	ECRP
MCB	**0.801**										
PCU	0.321	**0.807**									
TP	0.279	0.302	**0.831**								
OCL	0.203	0.183	−0.252	**0.799**							
IEL	−0.148	0.219	−0.346	0.202	**0.853**						
RCPS	0.353	−0.204	0.412	−0.183	−0.223	**0**.835					
ECF	−0.249	−0.198	−0.228	0.218	0.265	−0.205	**0.846**				
CuB	0.450	0.242	0.506	−0.202	−0.253	0.202	−0.302	**0.804**			
CoB	−0.223	0.291	−0.291	−0.184	−0.271	0.231	−0.338	0.231	**0.793**		
ST	0.181	0.217	−0.235	−0.253	−0.153	0.342	−0.257	−0.184	−0.216	**0.807**	
ECRP	−0.253	−0.308	−0.352	0.209	0.216	−0.403	−0.28	−0.265	−0.248	−0.232	**0.824**

The bold values are the square root of AVE.

### 4.3 Structural model and hypothesis testing

The current study validated 12 out of 13 proposed hypotheses (Table 5). Specifically, Hypothesis 5b (coefficient: 0.120) was not supported, indicating that IEL did not significantly impact confirmation bias (CoB). Hypothesis 1 (coefficient: 0.321, *p* < 0.001), Hypothesis 2 (coefficient: 0.282, *p* < 0.001), Hypothesis 3 (coefficient: 0.403, *p* < 0.001), Hypothesis 4c (coefficient: −0.256, *p* < 0.001), Hypothesis 6 (coefficient: 0.354, *p* < 0.01) and Hypothesis 8c (coefficient: −0.231, *p* < 0.001) were significantly supported, showing that the effects of MCB on CuB, PCU on CoB, TP on ST, OCL on ST, RCPS on ST and ST on ECRP are highly significant. Hypothesis 4a (coefficient: −0.209, *p* < 0.01), Hypothesis 7a (coefficient: −0.257, *p* < 0.01), Hypothesis 7b (coefficient: −0.206, *p* < 0.01), and Hypothesis 8a (coefficient: −0.182, *p* < 0.01) and Hypothesis 8b (coefficient: −0.219, *p* < 0.01) were supported, indicating that OCL impacted ST, ECF had a certain influence on both CuB and CoB, and ECRP is influenced by CuB and CoB. Finally, Hypothesis 4b (coefficient: −0.184, *p* < 0.05) and Hypothesis 5a (coefficient: −0.153, *p* < 0.05) were validated, illustrating that OCL impacted CoB and IEL influenced CuB.

**Table 5:** Path analysis results.

Hypothesis	Path	Coefficient	Deviation	*t*-value
H1	MCB → CuB	0.321***	0.052	6.432
H2	PCU → CoB	0.282***	0.051	5.631
H3	TP → ST	0.403***	0.043	8.071
H4a	OCL → CuB	−0.209**	0.048	4.035
H4b	OCL → CoB	−0.184*	0.051	3.607
H4c	OCL → ST	−0.256***	0.053	5.028
H5a	IEL → CuB	−0.153*	0.058	3.029
H5b	IEL → CoB	−0.120	0.068	1.432
H6	RCPS → ST	0.354***	0.054	7.028
H7a	ECF → CuB	−0.257**	0.057	5.025
H7b	ECF → CoB	−0.206**	0.052	4.036
H8a	CuB → ECRP	−0.182**	0.061	3.607
H8b	CoB → ECRP	−0.219**	0.059	4.251
H8c	ST → ECRP	−0.231***	0.053	4.629

***p < 0.001; **p < 0.01; and *p < 0.05.

**Figure 2:**
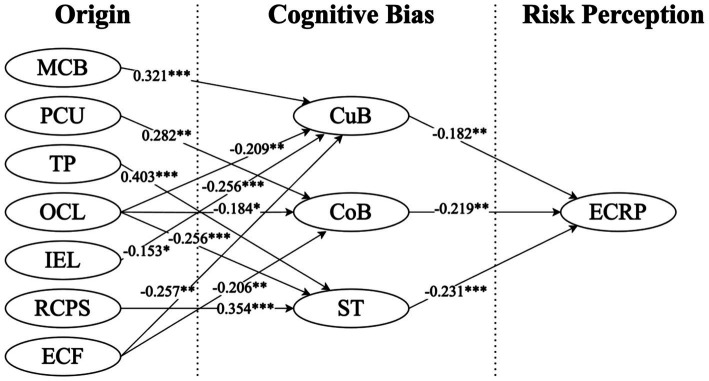
Path analysis results (Only supported paths were displayed). ***p < 0.001; **p < 0.01; and *p < 0.05.

All the supportive paths are illustrated in Figure 2.

## 5 Discussion

### 5.1 Impact of cognitive biases

According to the results of the data analysis, the average values for CuB, CoB, and ST are 3.54, 3.72, and 3.97, respectively. These figures indicate that such biases are not only prevalent among employees in international construction projects but also tend to be moderately high, particularly with ST being the most pronounced. These cognitive biases significantly influence employees’ ECRP, thereby highlighting the critical role of cognitive biases in shaping perceptions of environmental compliance risks. The impact of CuB arises from misunderstandings or misinterpretations of environmental compliance criteria across diverse cultural frameworks. For instance, practices like extensive excavation, viewed as advancement in certain cultures, may overlook its potential damage to groundwater systems, thereby threatening public health (Reichman and Seabloom, 2002). This practice might create confusion about cross-national environmental standards and public health requirements in international construction projects (Liu et al., 2018). CoB can cause individuals to disregard evidence that challenges their entrenched beliefs, such as domestically acceptable waste treatment methods being restricted in international settings due to potential harm to public health, yet habitual operations may lead some individuals to inadvertently ignore these international variations. ST compels a focus on immediate goals, potentially leading to compromised actions such as the improper disposal of hazardous chemical-laden industrial wastewater before deadlines or overlooking environmental impact assessments to meet project timelines, thereby increasing the risk of violating environmental compliance (Slawinski et al., 2017).

**Figure 3:**
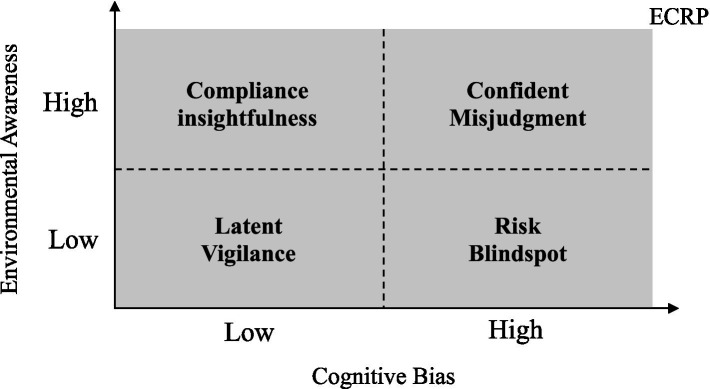
Quadrants of ECRP.

In conventional studies, environmental awareness is deemed a critical element shaping individual environmental compliance behaviors. When individuals possess a comprehensive understanding of environmental protection issues, they are more likely to develop appropriate environmental compliance attitudes (Nkonya et al., 2008; Okumah et al., 2018), which is often associated with precise environmental risk evaluations. However, this scenario becomes more complex in the context of international construction projects, where interactions with external information add layers to the intricacies of thought and cognition (Fee et al., 2013). A deficient perception of compliance risk among employees is not always attributable to inadequate compliance awareness. Cognitive biases can distort ECRP, leading to misguided actions. Integrating environmental awareness with cognitive biases as indicators for assessing ECRP unveils four potential perceptual states within individuals (Figure 3):

(1) Compliance Insightfulness reflects a state where individuals show minimal cognitive biases while demonstrating a strong awareness of environmental compliance, enabling nuanced risk assessments (Treviño et al., 1998). Such individuals are skilled at identifying a wide range of environmental compliance risks and implementing suitable mitigations. For example, in international construction projects, managers with compliance insightfulness excel in recognizing and interpreting environmental compliance differences due to cultural diversities, assessing their potential impact on project success.
(2) Confident Misjudgment indicates a state where individuals, convinced of their thorough understanding of environmental compliance risks, form their risk assessments based on misconceptions, biases, or inaccuracies, leading to inferior decisions (Philander, 2023; Robinson and Marino, 2015). A case in point is an experienced project manager whose cognitive biases may persuade his team that they can identify and manage all environmental compliance risks. Such overconfidence might cause them to overlook information that contradicts their beliefs, potentially resulting in unexpected difficulties due to the disregard of crucial risk factors (Hemmasi and Downes, 2013).
(3) Latent Vigilance characterizes a situation where individuals, despite an absence of overt cognitive biases, possess an incomplete understanding and insight into environmental compliance risks (Zhao and Qi, 2020). For instance, in international construction projects, managers might struggle to fully comprehend the regulatory frameworks and standards across different countries. While acknowledging the significance of compliance, the complexities of adapting to the distinct requirements of various legal jurisdictions might escape them, creating ambiguity or uncertainty that elevates risks associated with compliance.
(4) Risk Blindspot occurs when individuals show a significant deficiency in understanding environmental compliance requirements, further exacerbated by cognitive biases (Tversky and Kahneman, 1974; Zaiane and Ben Moussa, 2018). In international construction projects, such individuals might overlook critical environmental compliance mandates. For example, due to unfamiliarity, managers might underestimate the significance of local environmental regulations or, influenced by cognitive bias, deem certain requirements either irrelevant or minor to their projects, inadvertently heightening environmental compliance risks.

Four quadrants represent distinct groups of individuals. Concerning each group, international contractors should establish and refine unified strategies and measures at the organizational level, creating a systematic strategic framework. Throughout the various project stages, managers can assess the ECRP types of their subordinates using specialized evaluation scales. Finally, based on the company’s strategic framework, tailored interventions and training should be implemented to meet the specific needs of each employee group.

### 5.2 Origins of cognitive biases

This study reveals that IEL does not notably mitigate CoB. Contrary to the view that extensive international exposure deepens compliance adherence (Dahl, 2013; Kim and Kim, 2017), it may actually entrench cognitive patterns (Tversky and Kahneman, 1981), fostering overconfidence and a reluctance to assimilate disconfirming evidence (Schwind and Buder, 2012). Such ingrained views can lead to a biased focus on anticipated information in new contexts (Korteling et al., 2018; Toomey, 2023), rather than an open consideration of new or opposing information. Consequently, IEL could potentially amplify rather than reduce the propensity for CoB (Korteling et al., 2023; Rotman, 2012).

This study investigates key influencers on cognitive biases, revealing that CuB is significantly influenced by MCB, IEL, OCL, and ECF. CoB is mainly affected by PCU, OCL, and ECF, while ST is significantly impacted by TP, OCL, and RCPS. Traditional research often focuses on individual factors such as experience (Aberegg et al., 2005), cognitive ability (Foth, 2016), risk preference (Otuteye and Siddiquee, 2015) and gender (Hou et al., 2024), as well as social factors like institutional and social environments, and social interactions (Bertoldo et al., 2021; Joffe, 2003). Expanding on these views, this study proposes a tripartite causality framework incorporating individual, organizational, and project dimensions, tailored to the specifics of international construction environmental compliance. This multidimensional, hierarchical framework not only underscores the combined influence of individual and social factors on cognitive biases but also highlights the prominence of social factors through organizational and project dimensions in international construction projects. Identifying and effectively managing these dimensions is crucial for international contractors to refine cognitive bias mitigation and enhance environmental compliance management practices.

### 5.3 Managerial implications

**Figure 4:**
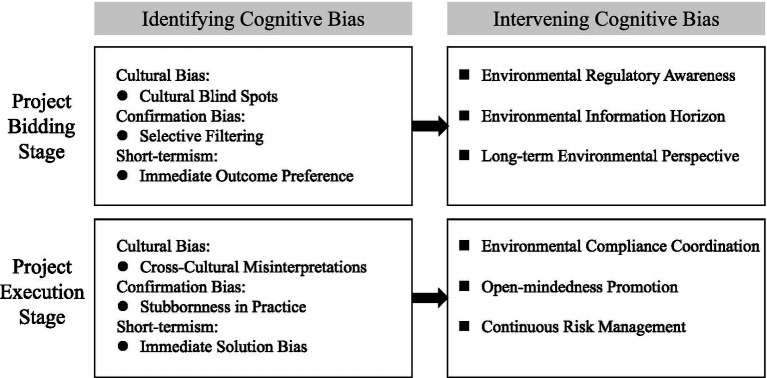
Identifying and addressing cognitive biases in bidding and execution phases.

The bidding and implementation phases are pivotal in the lifecycle of international construction projects (Kerzner, 2017). During the bidding phase, the focus of international contractors lies in accurately assessing project opportunities and devising effective strategies. The implementation phase, in contrast, centers on the execution of project plans and the ongoing management of risks (Project Management Institute, 2021). By identifying and addressing potential cognitive biases at these critical junctures, international contractors can significantly enhance decision-making quality, mitigate environmental compliance risks, and thus improve the overall success rate of projects. Psychological assessment tools, such as cognitive bias questionnaires (Gaasedelen et al., 2019; van der Gaag et al., 2013), and advanced data analysis techniques, like scenario simulation (Violato et al., 2021) and case study analysis (Wen et al., 2022), are instrumental in pinpointing specific manifestations of cognitive biases during these phases, thus enabling targeted interventions (Figure 4).

During the bidding phase, CuB manifests as Cultural Blind Spots, where personnel venturing into new markets might overlook the distinct environmental standards of different countries, mistakenly believing that environmental strategies successful in their own countries will be equally effective elsewhere. Moreover, the substantial differences in environmental laws and regulatory frameworks across nations pose challenges to employees’ comprehension of new compliance requirements, potentially leading to misunderstandings or neglect of local environmental laws and operational practices, thereby amplifying environmental compliance risks. CoB predominantly manifests as Selective Filtering, wherein employees confronted with an abundance of environmental assessments and impact reports might disregard or undervalue information that contradicts their initial expectations. This bias may lead to an overly optimistic environmental risk assessment, potentially overlooking threats to environment. ST is chiefly characterized by an Immediate Outcome Preference focus on immediate financial savings and quick project timelines, sidelining environmental sustainability. This approach often results in setting unrealistic project schedules and budget estimates, ignoring long-term environmental compliance risks. During the bidding process, the emphasis frequently lies solely on immediate gains. This perspective can lead to actions such as concealing environmental violations or leveraging misrepresented environmental credentials to secure contracts (Owusu et al., 2019), thereby neglecting the firm’s genuine ability to meet stringent environmental standards and manage environmental compliance risks effectively during the construction and operational phases.

During the implementation phase, CuB often emerge as Cross-Cultural Misinterpretations, particularly impacting environmental compliance aspects in team collaboration and daily interactions. Team members might misinterpret the expectations related to environmental protection measures and standards from colleagues of diverse cultural backgrounds, such as misreading signals related to environmental safeguards or encountering cultural clashes with local labor and suppliers over environmental and health issues. CoB in the context of environmental compliance manifests as Stubbornness in Practice, where employees find it challenging to adapt to new environmental regulations. Influenced by previous experiences in different regulatory contexts (Crocitto et al., 2005), team members may persist in using familiar methods and processes, even when they are ineffective in new work contexts, and could increase environmental pollution. Throughout the project, there is a tendency to rigidly adhere to the original plan, overlooking emerging environmental compliance issues and new information. This approach can lead to ignoring critical environmental impact assessments or insufficient evaluation of environmental compliance risks. ST primarily appears as Immediate Solution Bias, with some employees seeking quick fixes to environmental and health challenges, thereby sidelining the project’s quality, legality, and long-term sustainability goals. This practice may result in long-term negative impacts on the environment, for example, by implementing inadequate wastewater treatment measures or using harmful substances without fully assessing their potential harm to the environment.

During the bidding phase, three targeted interventions can effectively mitigate cognitive biases. First, enhancing Environmental Regulatory Awareness is essential. International contractors should provide specialized training on environmental compliance standards, covering diverse regional regulations and compliance requirements, with a focus on cross-cultural differences. These trainings should incorporate real-world case studies to help employees understand compliance risks and common issues across various regions. Additionally, contractors should consider inviting environmental compliance experts or consultants for regular workshops, ensuring that employees stay informed about the latest legal and regulatory developments. Involving compliance consultants in the bidding process can further ensure that environmental and public health considerations are fully integrated into bidding strategies, reducing the risk of non-compliance due to regional regulatory discrepancies. Second, broadening the Environmental Information Horizon is crucial. Employees should be encouraged to adopt a more comprehensive perspective when evaluating project information, challenging preconceived notions. This can be achieved through multidisciplinary risk assessment workshops that bring together expertise from project management, environmental compliance, and public health to identify and evaluate potential risks. These workshops should also simulate real-world project scenarios, allowing employees to practice addressing compliance challenges in a controlled setting. By promoting diverse viewpoints and critical thinking, these sessions can reduce confirmation bias and improve decision-making. Finally, fostering a Long-Term Environmental Perspective is vital. International contractors should establish an environmental sustainability committee to regularly evaluate the long-term environmental impacts of each project and incorporate these findings into bidding decisions. By conducting periodic reviews of past projects’ environmental performance, contractors can leverage these insights to refine future bidding strategies. Additionally, internal mechanisms should be created to balance short-term commercial goals with long-term sustainability objectives, ensuring that employees consider the enduring environmental and compliance risks alongside immediate project gains.

During the implementation stage, addressing potential cognitive biases with targeted interventions is crucial for ensuring environmental compliance. First, an Environmental Compliance Coordination strategy is essential to mitigate the adverse impacts of cultural and operational biases on environmental standards. This involves regular training sessions on global and local environmental regulations and best practices, fostering a shared understanding and commitment to environmental compliance among team members from diverse cultural backgrounds. Initiatives such as environmental impact assessment workshops and sustainability brainstorming sessions can enhance the team’s capacity to identify and address potential environmental risks effectively. Second, promoting Open-mindedness is key to overcoming biases towards traditional practices that may not align with contemporary environmental compliance requirements. Encouraging team members to explore and adopt green technologies and sustainable methods can lead to more environmentally friendly project outcomes. This approach not only challenges the status quo but also promotes a culture of continuous improvement and adaptation to emerging environmental standards. Finally, a Continuous Risk Management strategy is critical for maintaining a long-term focus on environmental compliance. This involves integrating ongoing environmental monitoring and risk assessment processes to swiftly identify and address potential environmental and health hazards. Implementing strategies for continuous improvement based on real-time data and feedback ensures that the project remains aligned with environmental compliance goals.

### 5.4 Theoretical implications

This study introduces a novel perspective on the implementation of environmental compliance management in international construction projects, providing a unique balance between theoretical insights and practical implications. Existing literature primarily focuses on the direct implementation of technical and management strategies (Prakash, 2001; Wang and Juo, 2021), as well as factors such as environmental awareness among individuals (Zsóka, 2008), to promote environmental compliance and sustainability. These studies often emphasize institutional frameworks, policy orientations, and specific environmental management technologies, overlooking the impact of project team members’ cognitive biases on ECRP.

Diverging from traditional research, this study explores the role of cognitive biases in the perception and management of environmental compliance risks, proposing an innovative framework that integrates cognitive psychology with environmental compliance management. The identification and intervention of cognitive biases are highlighted as key strategies for enhancing environmental compliance efforts, underscoring the importance of improving project teams’ perception abilities toward environmental compliance risks. This approach facilitates a comprehensive consideration of environmental protection.

By introducing the ‘Compliance Perception Quadrant’, the study innovatively integrates individuals’ cognitive biases regarding environmental compliance risks with their environmental awareness. This offers a new tool for assessing and enhancing project teams’ sensitivity and response capabilities towards environmental risks. This methodology surpasses the traditional focus on institutional and managerial perspectives, emphasizing the crucial role of psychological and cognitive factors in environmental compliance management.

Contrasting with other research, the theoretical contribution and innovation of this study lie in its exploration of the psychological dimensions of environmental compliance risk management. It proposes novel strategies for enhancing environmental compliance management capabilities in international construction projects through understanding and intervening in cognitive biases, thereby ensuring the realization of environmental standards. This research not only provides new theoretical perspectives and practical methods for the field of international construction project management but also highlights the importance of enhancing environmental compliance effectiveness from the perspective of cognitive biases.

This contribution enriches the body of knowledge on environmental compliance risk management in international construction projects, offering significant insights for academic research and practical application in related fields. It underscores the pivotal role of psychology in addressing environmental challenges, marking a significant step forward in the interdisciplinary approach to sustainable project management.

### 5.5 Limitations and future research

While this study offers valuable insights, it is not without its limitations. The use of snowball sampling predominantly involving Chinese contractors may affect the generalizability of the findings. Future research should aim to broaden the sample base by including contractors from a variety of national backgrounds to enhance the applicability and accuracy of the outcomes. Moreover, addressing cognitive biases presents a significant challenge. Although this study proposes strategies for mitigating cognitive biases among international construction practitioners, further exploration of a wider array of intervention strategies is needed. Incorporating a diverse range of methodological approaches could yield broader perspectives and deeper understanding, contributing to more effective solutions and improvements in managing cognitive biases.

## 6 Conclusion

This study explores the influencing factors of cognitive biases and their impact on ECRP in international construction projects, underscoring crucial role of cognitive biases in shaping environmental compliance risk assessment outcomes and the potential for misinterpretation. It emphasizes the vital necessity of integrating cognitive psychology principles into environmental compliance management practices in international construction projects, aiming to deepen the understanding of the importance of environmental compliance requirements for protecting the environment. Furthermore, the research advocates for the development of customized training programs and decision-support tools designed to mitigate the effects of cognitive biases. Such interventions seek to enhance the accuracy of environmental compliance risk assessments and strengthen the environmental compliance management capabilities of international contractors, thereby reducing negative impacts on the environment. This study not only significantly advances the theoretical understanding of ECRP and management but also highlights the practical value of cognitive psychology in enhancing environmental compliance. It proposes innovative ‘cognitive correction’ strategies to address the challenges faced in international construction compliance management, offering practical strategies and methods for the field of international construction project management.

## Data Availability

The raw data supporting the conclusions of this article will be made available by the authors, without undue reservation.
